# Supplementary material for: Cell-Free DNA Analysis by Whole-Exome Sequencing for Hepatocellular Carcinoma: A Pilot Study in Thailand
Source: Cancers (Basel). 2021 May 6;13(9):2229. doi: 10.3390/cancers13092229 (PMC8125351; doi:10.3390/cancers13092229)
Supplement: Supplementary file 1 [file cancers-13-02229-s001.zip › cancers-1177767-supplementary - figures.pdf]

Supplementary Materials: Cell-Free DNA Analysis by Whole-Exome Sequencing for Hepatocellular Carcinoma: A Pilot Study in Thailand

Pattapon Kunadirek, Natthaya Chuaypen, Piroon Jenjaroenpun, Thidathip Wongsurawat, Nutcha Pinjaroen, Pongserath Sirichindakul, Intawat Nookaew and Pisit Tangkijvanich

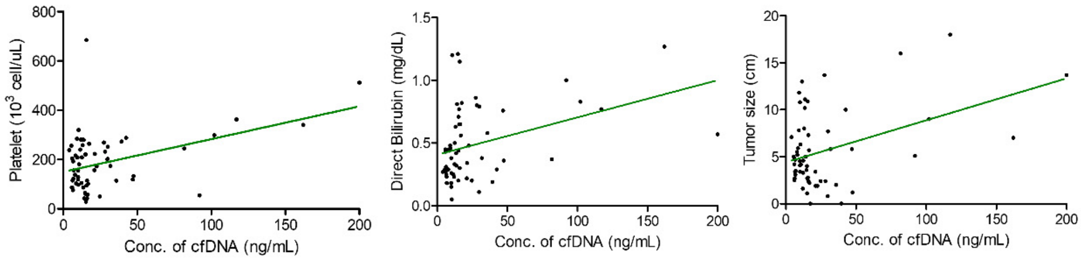

Figure S1. Pearson's correlation of cfDNA and clinical information on HCC patients.

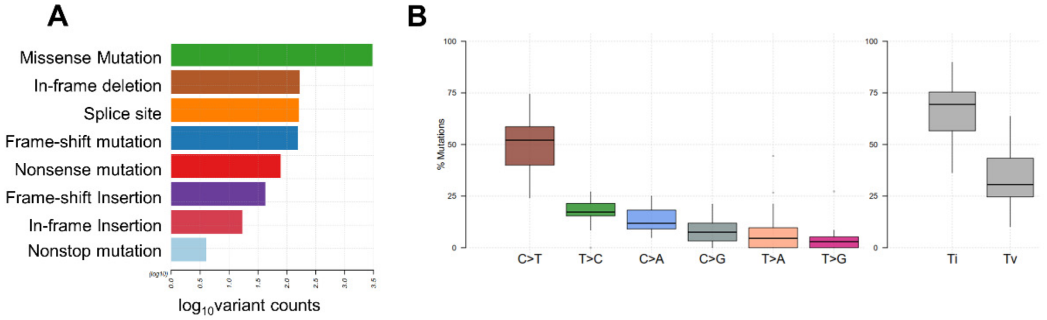

Figure S2. Summary of the mutational profiles of 30 HCC cfDNA samples.

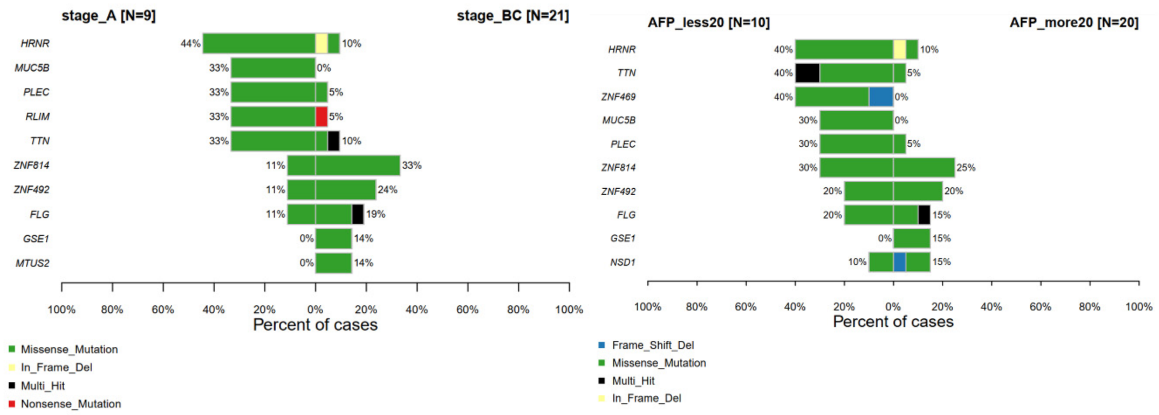

Figure S3. Distribution of early mutated genes. Patients with HCC were divided into early and intermediate stages (left). Patients with HCC were divided by AFP value (right). *HRNR*, *MUC5B*, *PLEC*, and *TTN* mutations were found frequently in early-stage HCC patients with a low AFP value.

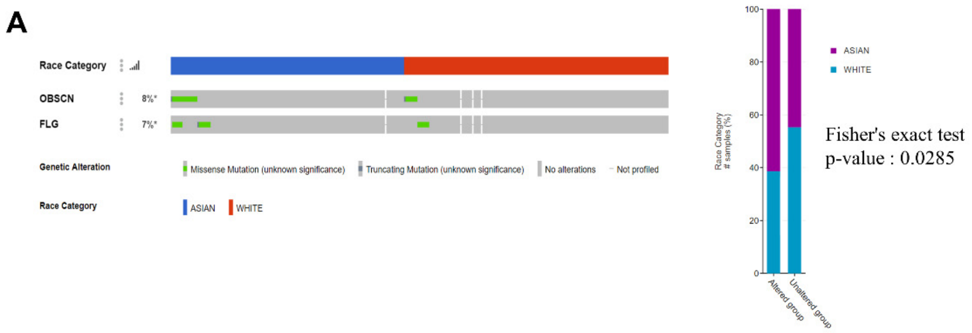

Figure S4. Comparison of *OBSCN* and *FLG* mutations in Asian and White individuals.

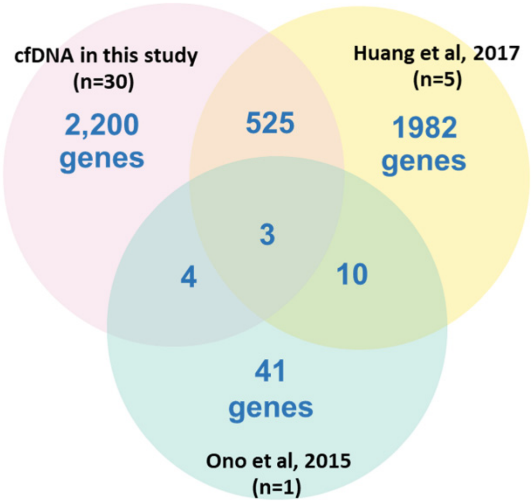

Figure S5. Comparison of SNVs in cfDNA identified with WES from this study and others.

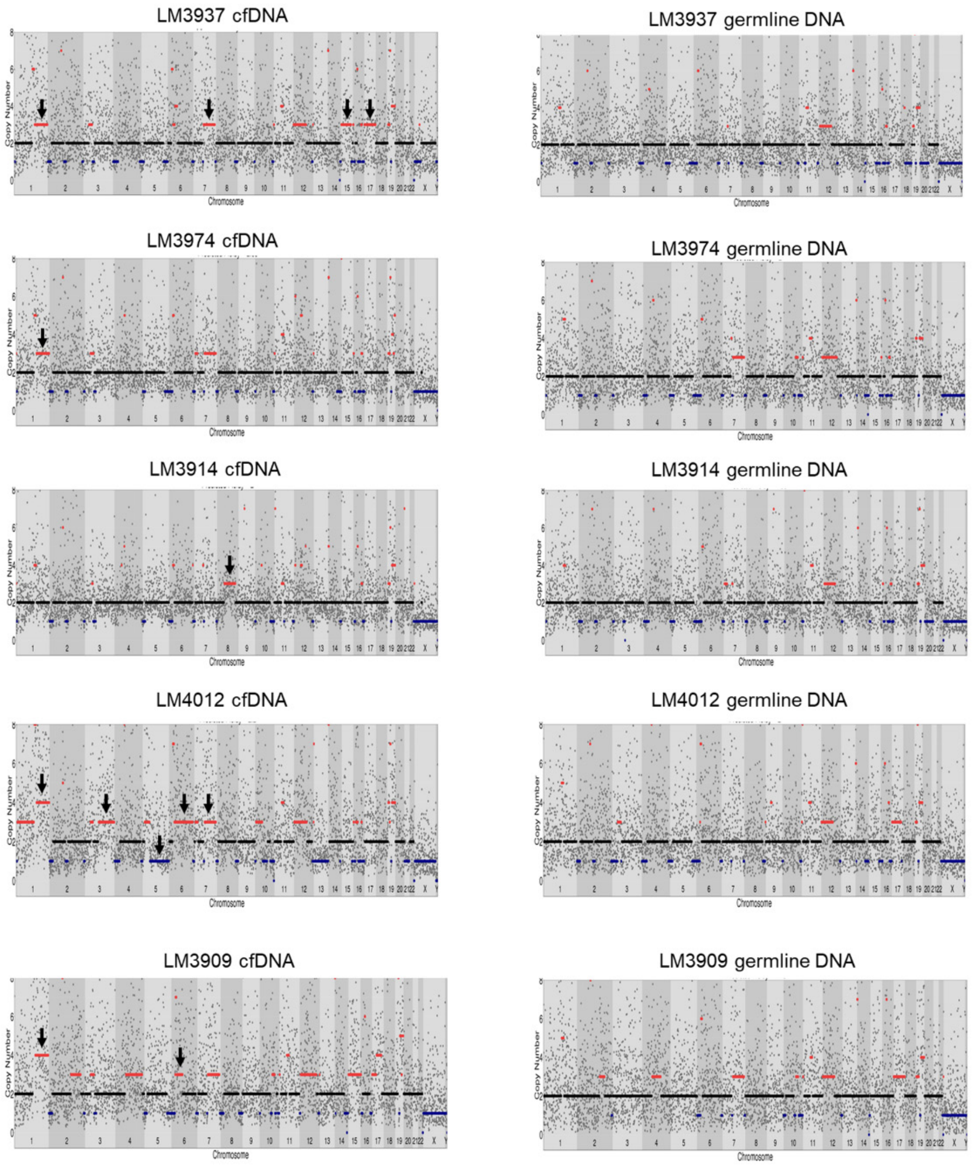

Figure S6. Copy number variation detection in cfDNA and germline DNA from LM 3937, LM3974, LM 4012, LM 3914 and LM 3909 using WES.

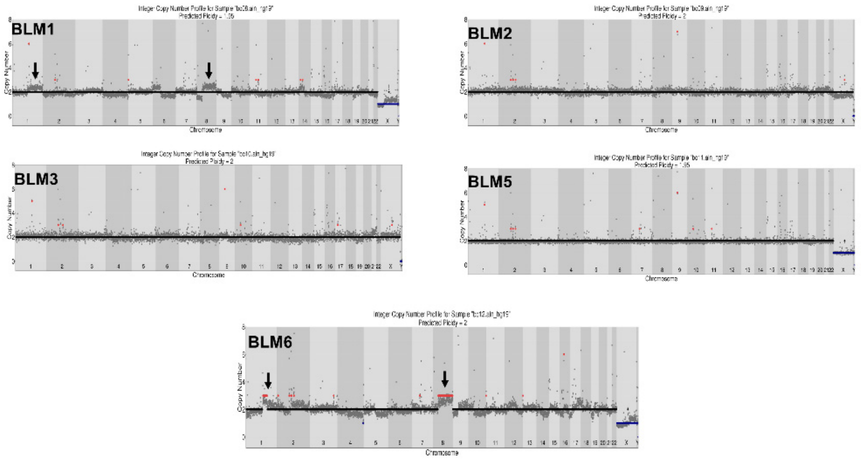

Figure S7. CNVs in HCC tumor DNA from SMURF-seq.

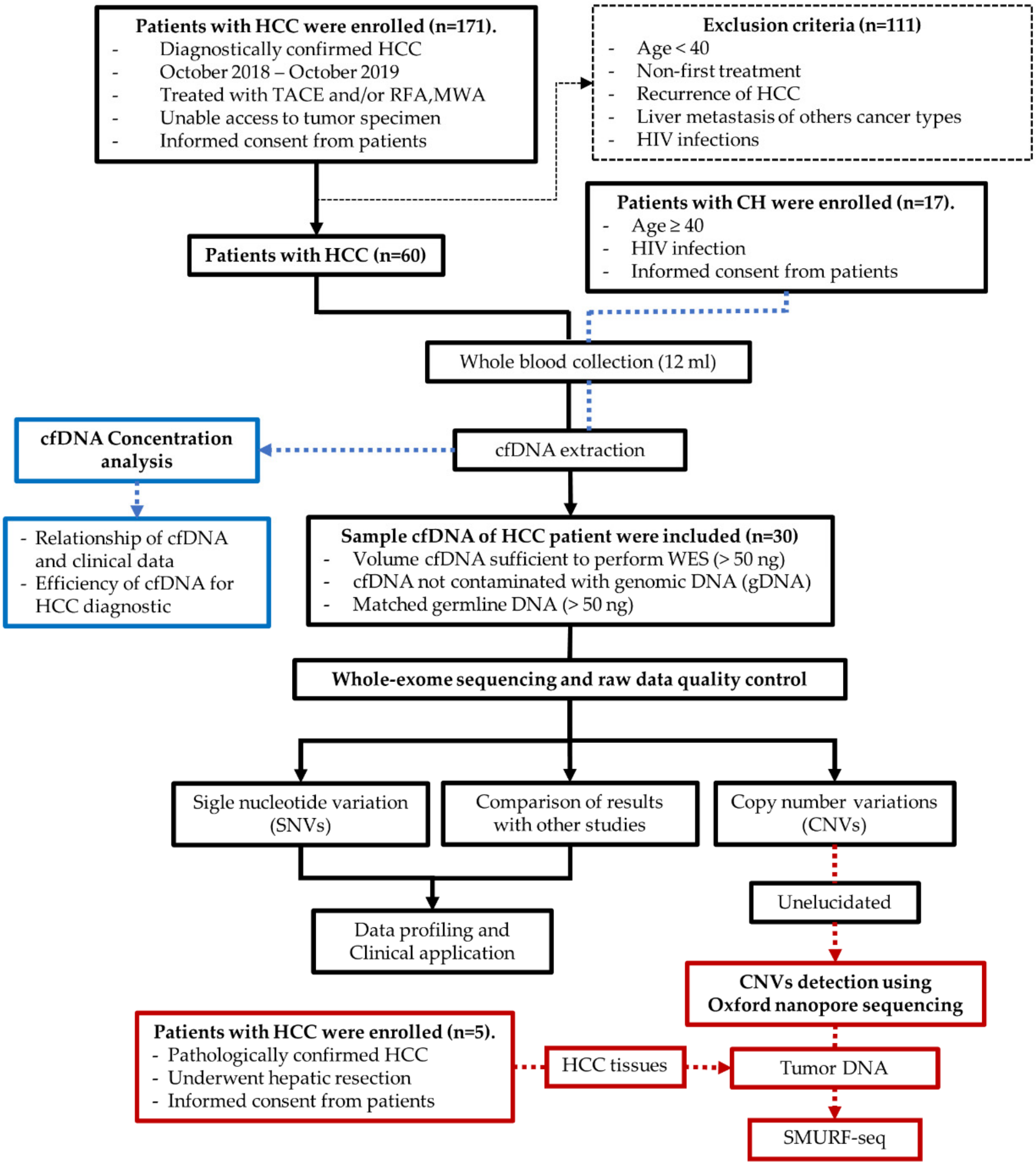

Figure S8. Overview of this study.
