# Supplementary material for: Cell-Free DNA Analysis by Whole-Exome Sequencing for Hepatocellular Carcinoma: A Pilot Study in Thailand
Source: Cancers (Basel). 2021 May 6;13(9):2229. doi: 10.3390/cancers13092229 (PMC8125351; doi:10.3390/cancers13092229)
Supplement: Supplementary file 1 [file cancers-13-02229-s001.zip › Supplementary_information.pdf]

# Supplementary code

## Trimmomatic: trimming of low quality read and adaptors

```
java -jar <path to trimmomatic.jar> PE [-threads <threads] [-phred33 | -phred64] [-trimlog
<logFile>] >] [-basein <inputBase> | <input 1> <input 2>] [-baseout <outputBase> |
<unpaired output 1> <paired output 2> <unpaired output 2> <step 1> ...
```

## Next flow (Sarek version 2.5.2): Preprocessing step

```
nextflow run nf-core-sarek-2.5.2/workflow -profile singularity -with-singularity nf-core-sarek-2.5.2.simg -with-
report -with-trace -with-dag flowchart.png –input input_file.tsv --max_memory xx.GB --max_cpus xx --tools
Haplotypecaller,Strelka,Mutect2,FreeBayes,mpileup,TIDDIT,Manta,ASCAT --acLoci '
1000G_phase3_20130502_SNP_maf0.3.loci' --acLociGC '1000G_phase3_20130502_SNP_maf0.3.loci.gc' --
bwaIndex 'human_g1k_v37_decoy.fasta.{amb,ann,bwt,pac,sa}' --chrDir '
/GATK/GRCh37/Sequence/Chromosomes' --chrLength '
/GATK/GRCh37/Sequence/Length/human_g1k_v37_decoy.len' --dbsnp
'GATK/GRCh37/Annotation/GATKBundle/dbsnp_138.b37.vcf' --dbsnpIndex
'GATK/GRCh37/Annotation/GATKBundle/dbsnp_138.b37.vcf.idx' --dict
'GATK/GRCh37/Sequence/WholeGenomeFasta/human_g1k_v37_decoy.dict' --fasta
'GATK/GRCh37/Sequence/WholeGenomeFasta/human_g1k_v37_decoy.fasta' --fastaFai
'GATK/GRCh37/Sequence/WholeGenomeFasta/human_g1k_v37_decoy.fasta.fai' --germlineResource
'GATK/GRCh37/Annotation/GermlineResource/gnomAD.r2.1.1.GRCh37.PASS.AC.AF.only.vcf.gz' --
germlineResourceIndex
'GATK/GRCh37/Annotation/GermlineResource/gnomAD.r2.1.1.GRCh37.PASS.AC.AF.only.vcf.gz.tbi' --intervals
'GATK/GRCh37/Annotation/intervals/wgs_calling_regions_Sarek.list' --knownIndels
'GATK/GRCh37/Annotation/GATKBundle/{1000G_phase1,Mills_and_1000G_gold_standard}.indels.b37.vcf' --
knownIndelsIndex
'GATK/GRCh37/Annotation/GATKBundle/{1000G_phase1,Mills_and_1000G_gold_standard}.indels.b37.vcf.idx' -
-snpeffDb 'GRCh37.75' --vepCacheVersion '95' --species 'homo_sapiens' --genome GRCh37 --exome --targetBED
GRCh37_S07604514_Regions.bed
```

## Create Somatic Panel of Normal

**Step 1.** Run Mutect2 in tumor-only mode for each normal sample.

```
gatk Mutect2 -R human_g1k_v37_decoy.fasta -I input_file.bam -max-mnp-distance 0 -O output_file.vcf.gz
```

**Step 2.** Create a GenomicsDB from the normal Mutect2 calls.

```
gatk GenomicsDBImport -R human_g1k_v37_decoy.fasta -L wgs_calling_regions_Sarek.list --genomicsdb-
workspace-path pon_db -V input_file.vcf.gz
```

**Step 3.** Combine the normal calls using CreateSomaticPanelOfNormals.

```
gatk CreateSomaticPanelOfNormals -R human_g1k_v37_decoy.fasta -V gendb://pon_db -O pon.vcf.gz
```

## Mutect2: somatic mutation calling in tumor-normal mode

**Step 1.** Mutect2

gatk Mutect2 -R human_g1k_v37_decoy.fasta -I input_tumor_file.recal.bam -tumor LM3858cf -I input_normal_file.recal.bam -normal LM3858gl --panel-of-normals pon.vcf.gz -O output_Mutect2_unfiltered.vcf.gz --f1r2-tar-gz f1r2.tar.gz --native-pair-hmm-threads xx

**Step 2.** Learn Read Orientation Model

gatk LearnReadOrientationModel -I f1r2.tar.gz -O read-orientation-model.tar.gz

**Step 3.** Get Pileup Summaries of tumor and normal

gatk GetPileupSummaries -I input_tumor_file.recal.bam -V gnomAD.r2.1.1.GRCh37.PASS.AC.AF.only.vcf.gz -L wgs_calling_regions_Sarek.list -O output_tumor_file _pileups.table

gatk GetPileupSummaries -I input_normal_file.recal.bam -V gnomAD.r2.1.1.GRCh37.PASS.AC.AF.only.vcf.gz -L wgs_calling_regions_Sarek.list -O output_normal_file _pileups.table

**Step 4.** Calculate Contamination

gatk CalculateContamination - input_tumor_file _pileups.table -matched input_normal_file _pileups.table -tumor-segmentation segments.table -O calculatecontamination.table

**Step 5.** Filter Mutect Calls

gatk FilterMutectCalls -R human_g1k_v37_decoy.fasta -V input_ Mutect2_unfiltered.vcf.gz --tumor-segmentation segments.table --contamination-table calculatecontamination.table --ob-priors read-orientation-model.tar.gz -O output_Mutect2_Filtered.vcf

**Funcotator: Variants annotation**

**Step 1.** Data Source Downloader
gatk FuncotatorDataSourceDownloader --somatic --validate-integrity --extract-after-download

**Step 2.** Filtter pass
vcftools --vcf input_Mutect2_Filtered.vcf --remove-filtered-all --recode --out pass_input_Mutect2_Filtered

**Step 3.** Running Funcotator in the GATK With Base Options
gatk Funcotator --variant pass_input_Mutect2_Filtered.recode.vcf --reference human_g1k_v37_decoy.fasta --ref-version hg19 --data-sources-path funcotator_dataSources.v1.6.20190124s --output output_annotated_variants.funcotated.vcf --output-file-format VCF

**cnvkit: Copy number analysis**

**Step 1.** Unmarking of duplicate read

gatk UnmarkDuplicates -I .recal.bam -O .unmarked.bam

**Step 2.** Splitting of target file

cnvkit.py target GRCh37_S07604514_Regions.bed --split -o GRCh37_S07604514_Regions_eq.bed

**Step 3.** CNV calling

cnvkit.py batch input_tumor.unmarked.bam --normal input_normal.unmarked.bam --targets GRCh37_S07604514_Regions_eq.bed --fasta human_g1k_v37_decoy.fasta --output-reference output.cnn --output-dir pathToOutput/ --diagram --scatter -p xx --drop-low-coverage -m hybrid --annotate refFlat.txt -y -c

**Step 4. Preparing file for ginkgo**

samtools view -hb  intput_normal.unmarked.bam | bedtools bamtobed -i stdin | gzip > output_normal.unmarked.bed.gz
samtools view -hb  input_normal.unmarked.bam | bedtools bamtobed -i stdin | gzip > output_normal.unmarked.bed.gz

**Nanopore sequencing analysis**

**Step 1.** Guppy base caller on GPU

guppy_basecaller -r -i path/to/input/fast5 -s path/to/output/fastq --kit code_ont_kit--flowcell code_ont_flowcell -x "cuda:0 cuda:1" --disable_pings --qscore_filtering --min_qscore 8

**Step 2.** Guppy barcoder

guppy_barcoder -t xx -r -I path/to/pass/fastq -s path/to/output --barcode_kits kit code_ont_barcode_kit

**Step 3.** Mapping with human genome and sorting

minimap2 -t XX -a -x map-ont /Homo_sapiens/UCSC/hg19/Sequence/WholeGenomeFasta/genome.fa path/to/fastqfile "|" samtools sort -@XX -o output_aligned.sorted.bam ";" samtools index output__aligned.sorted.bam

**Step 4.** Preparing file for ginkgo: Convert file bam file to bed file

samtools view -hb  input_aligned.sorted.bam | bedtools bamtobed -i stdin | gzip > output_aligned.bed.gz
